# Capturing the Context of Maternal Deaths from Verbal Autopsies: A Reliability Study of the Maternal Data Extraction Tool (M-DET)

**DOI:** 10.1371/journal.pone.0014637

**Published:** 2011-02-07

**Authors:** Ann L. Montgomery, Shaun K. Morris, Rajesh Kumar, Raju Jotkar, Prem Mony, Diego G. Bassani, Prabhat Jha

**Affiliations:** 1 Centre for Global Health Research, Li Ka Shing Knowledge Institute, St. Michael's Hospital, Toronto, Canada; 2 Division of Infectious Diseases, Hospital for Sick Children, Department of Pediatrics, University of Toronto, Toronto, Canada; 3 School of Public Health, Post Graduate Institute of Medical Education Chandigarh, Chandigarh, India; 4 St John's Research Institute, St. John's National Academy of Health Sciences, Bangalore, India; 5 Dalla Lana School of Public Health, University of Toronto, Toronto, Canada; Harvard Medical School, United States of America

## Abstract

**Background:**

The availability of quality data to inform policy is essential to reduce maternal deaths. To characterize maternal deaths in settings without complete vital registration systems, we designed and assessed the inter-rater reliability of a tool to systematically extract data and characterize the events that precede a nationally representative sample of maternal deaths in India.

**Method/Principal Findings:**

Of 1017 nationally representative pregnancy-related deaths, which occurred between 2001 and 2003, we randomly selected 105 reports. Two independent coders used the maternal data extraction tool (questions with coding guidelines) to collect information on antenatal care access, final pregnancy outcome; planned place of birth and care provider; community consultation, transport, admission, hospital referral; and verification of cause of death assignment. Kappa estimated inter-rater agreement was calculated and classified as poor (K≤0.4), moderate (K = 0.4-≤0.6), substantial (K = 0.6-≤0.8) and high (K>0.8) using the criteria from Landis & Koch. The data extraction tool had high agreement for gestational age, pregnancy outcome, transport, death *en route* and admission to hospital; substantial agreement for receipt of antenatal care, planned place of birth, readmission and referral to higher level hospital, and whether or not death occurred in the intrapartum period; moderate to substantial agreement for classification of deaths as direct or indirect obstetric deaths or incidental deaths; moderate agreement for classification of community healthcare consultation and total number of healthcare contacts; and poor agreement for the classification of deaths as sudden deaths and other/unknown cause of death. The ability of the tool to identify the most-responsible-person in labour varied from moderate agreement to high agreement.

**Conclusions:**

This data extraction tool achieved good inter-rater reliability and can be used to collect data on events surrounding maternal deaths and for verification/improvement of underlying cause of death.

## Introduction

The United Nation's Millennium Development Goal Number 5 targets a 75% reduction in the maternal mortality ratio by 2015 through Safe Motherhood Initiatives. The World Health Organization estimates that 99% of the approximately 300 000 annual maternal deaths occur in low- and middle-income countries [1]. India accounts for one-quarter of the maternal deaths worldwide [1]–[3] and depending on the state of residence, wealth quintile, urban/rural residence, and caste; there are large variations in the proportion of women receiving Indian Safe Motherhood Initiatives such as antenatal care (National average 15%, Range between States 34.3–99.9%), institutional delivery (38.7%, 10.4–93.1%), skilled birth attendance (46.6, 12.4–99.4%), and postnatal care (41.2%, 10.6–87.2%) [2], [4].

Maternal deaths are difficult to count because they are relatively rare events, and are prone to misclassification and under-reporting. Maternal mortality estimates are further hindered when no routine vital registration system is available and when the majority of deaths occurs outside of the healthcare system and without medical assignment of the cause of death [5]. However, evidence is essential to monitor trends in maternal health [6]–[9].

Proper characterization of maternal deaths requires the examination of the cause of death in the context of the reproductive event. The wider context in which a woman dies needs to be defined in order to determine the temporal relationship of the death within the pregnancy and in relation to the medical complication. As well, there is a need for improved data quality for differentiating direct and indirect maternal deaths [10], and monitoring indicators such as the most-responsible-person in labour (unskilled or skilled birth attendant) and planned place of birth (versus actual place of birth).

A verbal autopsy is a semi-structured interview carried out with a family member of the deceased. Questions are posed to elicit signs and symptoms of the final illness, health history, and events surrounding the death; this information is used to assign a probable underlying cause of death (i.e. the disease or injury that initiated the train of events leading directly to death). These verbal autopsy interviews can be incorporated in data-collection systems (demographic surveillance sites, sample registrations systems, censuses or household surveys). Verbal autopsies are beneficial where access to and uptake of healthcare is low, as they can be administered within the community [11].

The Registrar General of India, in collaboration with the Centre for Global Health Research, has implemented an enhanced form of verbal autopsies with its Sample Registration System (SRS) to monitor all deaths for 2001–2003. Details about the events that preceded the deaths are collected using a validated verbal autopsy tool called RHIME (Routine, Reliable, Representative, and Resampled Household Investigation of Mortality with Medical Evaluation) consisting of family informant responses to structured questions and an open-ended narrative about events preceding the death [12]–[15]. Details of earlier results have be published elsewhere [16]–[19]. For pregnancy-related deaths, an adult form and a maternal form is completed, and generates data on age, health history, gestational age, antenatal care, place of delivery, attendant at delivery, and symptoms around the time of death in addition to an open narrative. Two independent physicians review the questionnaires and verbal autopsy narrative and assign a cause of death using the International Classification of Disease 10th edition (ICD-10) [13], [18], [20]. If the physicians disagree on the cause of death there is a reconciliation and, if required, adjudication process in place.

The pregnancy-related deaths represent only a small proportion of all cause mortality (<1%) in the MDS. It is not be feasible to add new questions to the upcoming fieldwork future questionnaire or to the fieldworker training due to the overall small number of cases. For data already collected in the 2001–2010 period, there is a substantial amount of information in addition to the physician-assigned cause of death which until now has not been adequately extracted or assessed. Furthermore, it tends to be the RHIME narratives, not the short answer response that contains information on the planned place of birth (versus actual place of birth from the response to the short answer question *Where was the delivery?*), initial care provider (versus *Who attended the delivery?*), community consultation, transportation to hospital, hospital admission, referral to secondary hospital, death *en route*, and number of healthcare contacts. Finally, assigning cause of death for the 2001–2003 cases by two independent physicians was conducted using 2004 MDS guidelines. In anticipation of changes by WHO of ICD-11 in 2015, we refined the 2004 guidelines to take into account these upcoming changes to the classification. The objective of this study is to determine the inter-rater reliably of a data extraction tool to systematically code the events associated with pregnancy-related deaths, and guide the assignment of ICD-10 cause of death using 2011 guidelines.

## Methods

The data presented here come from a sample of 1017 maternal deaths identified in the MDS between 2001 and 2003. A structured maternal questionnaire was added to the RHIME following the initial project implementation. All maternal narratives were translated from twelve different languages (Assamese, Bengali, Gujarati, Hindi, Kannada, Malayalam, Marathi, Oriya, Punjabi, Tamil, Telugu, and Urdu) into English. We used the WHO definition of maternal deaths, direct maternal death resulting from obstetric complication, and indirect maternal deaths resulting from diseases exacerbated by the pregnancy [1].

The MDS Maternal Data Extraction Tool (M-DET) is composed of two parts: (a) identifying discrete events that span pregnancy to postpartum and (b) verify ICD-10 cause of death. The M-DET consists of 9 main questions with 63 sub-questions used to direct systematic coding of the RHIMEs in four areas: (i) receipt of antenatal care and outcome of the final pregnancy; (ii) planned place of birth and care provider; (iii) consultation, transport, hospital admission, referral and number of healthcare contacts and (iv) underlying cause of death (Table 1).

**Table 1:** Summary of topic areas of M-DET.

Section	Topic	Details
i)	Antenatal care	Receipt of antenatal care at any point in the pregnancy
	Outcome of this pregnancy	Abortion, remained pregnant at time of death, vaginal delivery, cesarean delivery
ii)	Planned place of abortion/birth	Home, community, health facility
	Primary care provider	Trained/untrained traditional birth attendant, midwife, allopathic doctor, nonallopathic doctor, village ladies, no one, or not applicable (complication arose prior to the onset of labour)
iii)	Consultation	Consultation in community for complication
	Transport	Transport to hospital indicated for complication
	Death *en route*	Did the woman die *en route* to hospital?
	Admission	Was the woman admitted to hospital either for a planned hospital birth or the complication?
	Readmission	Was this a readmission following a hospital birth or follow-up following admission for same complication?
	Referral	Was the woman referred to another hospital?
	Healthcare contact	How may healthcare contacts did the woman have?
	Where did the woman die	Home, *en route* to hospital, in hospital, *en route* to referral hospital, at referral hospital, at home following discharge from hospital
iv)	Underlying cause-of-death	
	Direct	Obstetric hemorrhage: Abortive outcome, Antenatal, Intrapartum/postpartum
	Maternal sepsis: Septic abortion, Postpartum sepsis, Obstetric tetanus	
		Hypertensive disorders of pregnancy
		Postpartum suicide
	Other/Unknown - probably obstetric condition	
	Indirect	Tuberculosis
		Other infection
		Other medical condition
		Antenatal Suicide
	Other/Unknown - probably nonobstetric condition	
	Incidental	Domestic violence
		Other

Ten per cent (n = 105) of cases were selected by simple random selection from the 1017 pregnancy-related deaths. Sample size was calculated for the minimum number of cases required in a two-rater study to detect a statistically significant Kappa (p≤0.05) with 90% power, assuming a null hypothesis of Kappa = 0.4. A physician (SKM) and a midwife (ALM) received simultaneous training for approximately three hours, comprised of review of the M-DET questions and standards for interpretation of information, common lexicon of terms used in local languages, and defined criteria for planned home versus hospital birth, initial care provider, community consultation, transport, hospital admission, referral and number of health care contacts. Coders were trained in ICD-10 coding, and provided with criteria of obstetric cause of death (“O-codes”) and were directed to verify MDS MD coded deaths for further quality assurance. The MDS has been restricted to 3-digit, and not 4-digit, ICD-10 coding of cause of death. The 4-digit cause of death provides further differentiation within a category. The coders were trained in defined criteria for further refinement of some causes of death by differentiating between septic versus hemorrhagic complications from abortion or miscarriage (O03-O06 with addition of .1 or .5); eclampsia in pregnancy, labour or postpartum (O15 with addition of .0, .1 or .2); and differentiation between septicemia in labour versus unspecified complications of labour (O75.3 versus O75.9). They then independently reviewed the short answer responses and narratives, and the cause of death assigned by the MDS physicians and used the M-DET to extract information on the four areas described above. Coders were instructed to take the narrative as the standard if there was a contradiction between the short answer response and the narrative. Inter-rater reliability was estimated with the weighted and unweighted Kappa statistic of nominal categorical and ordered categorical variables respectively, and 95% confidence intervals were calculated using bootstrap estimation of standard error for Kappa. The Landis and Koch classification of inter-rater reliability was used to interpret the coefficients: Kappa ≤0.4 - poor to fair agreement; >0.4 ≤0.6 - moderate agreement; >0.6 ≤0.8 - substantial agreement; >0.8 - high agreement [20], [21]. Microsoft Excel was used for data entry and Stata SE 10 (Stata, http//www.stata.com) was used for Kappa analysis and confidence interval calculation.

Kappa for variables derived from objective questions (antenatal care, cesarean delivery, place of birth) were calculated including and excluding these cases (missing questions) and there was no significant difference in Kappa values (data not shown).

Ethics approval for the MDS was obtained from School of Public Health, Post Graduate Institute of Medical Education, Chandigarh India; St John's Research Institute, St. John's National Academy of Health Sciences, Bangalore India; and St. Michael's Hospital, Toronto Canada.

## Results

### Access to antenatal care and pregnancy outcome

There was substantial agreement between coders for receipt of antenatal care (Kappa = 0.76, 0.62–0.85), whether the maternal death occurred in the intrapartum period (onset of miscarriage/labour within 24 hours following delivery/passing of the products of conception) (K = 0.70, 0.59–0.82), and whether the woman died in the postpartum period (between 24 hours of delivery and 42 days postpartum) (K = 0.70, 0.54–0.82). There was high agreement for gestational age (in months) at time of delivery or death (K = 0.94, 0.88–0.99); outcome of pregnancy: abortion (K = 1.00) or undelivered at death (K = 0.87, 0.72–0.95); whether the complications arose in the pregnancy prior to delivery (K = 0.94, 0.82–1.00); and mode of delivery for spontaneous vaginal delivery (K = 0.90, 0.83–0.98) or cesarean (K = 0.81, 0.55–1.00). For deliveries after 7 months gestation, there was high agreement for identification of live births (K = 0.83, 0.71–0.90) and still births (K = 0.83, 0.71–0.91) (Table 2).

**Table 2:** Unweighted Kappa statistic for access to antenatal care and outcome of final pregnancy (n = 105) (*weighted Kappa).

Description	Kappa	(95% Confidence Interval)
Report of receipt of antenatal care in the pregnancy	0.76	(0.62–0.85)
Gestational age at time of delivery or death*	0.94	(0.88–0.99)
Abortion	1.00	(1.00–1.00)
Pregnant at onset of complication without abortion or labour	0.94	(0.82–1.00)
Spontaneous vaginal delivery	0.90	(0.83–0.98)
Cesarean delivery	0.81	(0.55–1.00)
Intrauterine fetal death reported prior to woman's death	1.00	(1 .00–1.00)
Livebirth	0.83	(0.71–0.90)
Stillbirth	0.83	(0.71–0.91)
Woman died pregnant/undelivered	0.87	(0.72–0.95)
Woman died in the intrapartum period (in labour or <24 hours postpartum)	0.70	(0.59–0.82)
Woman died in the postpartum period	0.70	(0.54–0.82)
Number of days postpartum/postabortion*	1.00	(1.00–1.00)
Late postpartum death (>42 days postpartum <1year)	1.00	(1.00–1.00)
Number of months postpartum/postabortion*	1.00	(1.00–1.00)

### Planned of place birth or abortion and most-responsible person in attendance in labour/abortion

‘Planned place of birth’ was defined as the location where the woman and her family intended for her to deliver. Within this category, therapeutic abortions were classified as facility-based or non-facility-based. There was substantial agreement between coders in identifying planned home and planned hospital births for term delivery, and facility-based care for therapeutic abortion (K = 0.79, 0.69–0.90).

Overall, M-DET had substantial to high agreement in identifying the most-responsible-person to attend the labour/abortion. This information was reported to interviewers by the respondent and refers to the person who initially attended the woman in labour, or who performed the abortion. The exceptions (moderate agreement) were: (i) the identification of the attending physician as allopathic (K = 0.74, 0.62–0.95) or non-allopathic (K = 0.56, 0.33–0.72); (ii) identification of non-professional attendants (K = 0.58, 0.35–0.77), for “no one in attendance” and K = 0.65 (0.46–0.83) for “family or village ladies”); and (iii) when the complication developed before term and without having started labour (i.e. initial contact was for a complication and not delivery or therapeutic abortion) (K = 0.64, 0.47–0.81) (Table 3).

**Table 3:** Unweighted Kappa statistic for planned place of birth and most-responsible-person in labour (n = 105).

Description	Kappa	(95% Confidence Interval)
Planned place of birth or abortion: home or health facility	0.79	(0.69–0.90)
Trained Traditional Birth Attendant	0.98	(0.94–1.00)
Untrained Traditional Birth Attendant	0.94	(0.87–1.00)
Nurse/midwife	0.79	(0.64–0.88)
Allopathic Doctor	0.74	(0.62–0.95)
NonAllopathic doctor	0.56	(0.33–0.72)
Family/“Village ladies”	0.65	(0.46–0.83)
No one	0.58	(0.35–0.77)
Was pregnant at time of complication (prior to the onset of normal labour at term or therapeutic abortion)	0.64	(0.47–0.81)

### Consultation, transport, hospital admission and referral

There was high agreement in the identification of urgent transport from home to hospital, (K = 0.80, 0.71–0.88), as well as identification of death *en* route to hospital (K = 0.83, 0.70–0.91) and identification of outcome following admission to hospital (discharge, death and referral) (K = 0.89, 0.81–0.97). There was substantial to high agreement for the identification of admission (K = 0.87, 0.77–0.96) and readmission (K = 0.76, 0.62–0.87) to hospital, and referral to another hospital (K = 0.78, 0.58–1.00). Extraction of information about the total number of healthcare contacts during pregnancy (0, 1 and ≥2) achieved moderate reliability (K = 0.57, 0.45–0.75) (Table 4).

**Table 4:** Unweighted Kappa statistic for consultation, transport, admission and referral to hospital (n = 105).

Description	Kappa	(95% Confidence Intervals)
Consult home/community with healthcare worker for the complication	0.58	(0.42–0.71)
Transport for complication	0.80	(0.71–0.88)
Woman dies *en route* to hospital	0.83	(0.70–0.91)
Woman admitted to hospital	0.87	(0.77–0.96)
Outcome following admission	0.89	(0.81–0.97)
Woman was readmitted to hospital for complication	0.76	(0.62–0.87)
Woman was referred to higher hospital	0.78	(0.58–1.00)
Total number of healthcare contacts	0.57	(0.45–0.75)

### Underlying cause of death

Three cases were missing physician-assigned cause of death.

The two coders assigned an underlying cause of death using ICD-10 and the 2011 guidelines for maternal deaths and verbal autopsy. Deaths were classified under broad categories of direct or indirect maternal deaths, or incidental death. Direct obstetric deaths were obstetric hemorrhage, sepsis, hypertensive disorders of pregnancy, postpartum suicide, and other/unknown obstetric death. Obstructed labour was a contributory cause and was further assigned to a more specific mutually exclusive category of hemorrhage (uterine atony or uterine rupture), sepsis or unknown. Indirect obstetric deaths were tuberculosis, other infections, other medical conditions, antenatal suicide, or unknown/probably non-obstetric. Incidental deaths contains a subcategory of domestic violence (death by beating or kitchen fire) [21] (see Table 5).

**Table 5:** Unweighted Kappa for cause-of-death (n = 105).

Description	Kappa (95% Confidence Interval)
**Direct maternal death**	
Hemorrhage inclusive	0.76 (0.66–0.86)
Abortion hemorrhage	0.91 (0.82–0.98)
Antepartum hemorrhage	0.89 (0.82–0.98)
Postpartum hemorrhage	0.86 (0.77–0.93)
Sepsis or postpartum tetanus inclusive	0.56 (0.31–0.80)
Septic Abortion or miscarriage	1.00 (1.00–1.00)
Postpartum sepsis	0.80 (0.66–0.91)
Obstetrical tetanus	0.92 (0.80–1.00)
Hypertensive disorders of pregnancy	0.85 (0.57–1.00)
Postpartum suicide	1.00 (1.00–1.00)
Other/Unknown, probably obstetric cause	0.73 (0.60–0.82)
Indirect maternal deaths, inclusive	0.88 (0.70-1.00)
TB complicated by pregnancy	1.00 (1.00–1.00)
Other, infectious	0.95 (0.79–1.00)
Other, medical condition	0.95 (0.84–1.00)
Suicide	1.00 (1.00–1.00)
Other/Unknown, probably non-incidental, probably non-obstetric cause	0.00 (.- 1.00)
**Incidental inclusive**	1.00 (1.00–1.00)
Beating	1.00 (1.00–1.00)
Burning	1.00 (1.00–1.00)

#### Direct obstetric deaths

The use of M-DET for the identification of hemorrhage, including all obstetric hemorrhages, achieved substantial inter-rater agreement (K = 0.76, 95%CI 0.66–0.86). The following subcategories had high agreement: abortion/miscarriage (K = 0.91, 0.82–0.98), antepartum hemorrhage (K = 0.89, 0.82–0.98) and intrapartum/postpartum hemorrhage (K = 0.86, 0.77–0.93). Identification of deaths due to sepsis had moderate agreement (K = 0.56, 0.31–0.80) and the subcategories of septic abortion (1.00), postpartum sepsis (K = 0.80, 0.66–0.91) and obstetric tetanus (K = 0.92, 0.80–1.00) had high agreement. There was also high agreement for hypertensive disorders of pregnancy (K = 0.85, 0.57–1.00) and postpartum suicide (K = 1.00). ‘Other/Unknown’ category, where the underlying cause was deemed to be probably obstetric, had substantial agreement (K = 0.73, 0.60–0.82).

#### Indirect obstetric deaths and Incidental deaths

Using M-DET, coders could reliably identify indirect causes (K = 0.88, 0.70–1.00), including tuberculosis (K = 1.00), other infections (K = 0.95, 0.79–1.00) and other medical condition (K = 0.95, 0.84–1.00) and antenatal suicide (K =  1.00). Classification of the single category of “Other/unknown, non-incidental and probably non-obstetric” had no agreement for the 2 cases identified by one coder. Inter-rater agreement on incidental deaths was high (K = 1.00).

## Discussion

Overall, coders using the M-DET to extract information about events preceding a maternal death from the verbal autopsies obtained substantial to high inter-rater agreement. The MDS is designed to be a comprehensive but not extensively detailed cause of death study [13] and this tool extends the use of the RHIMEs for detailed studies of maternal mortality. The M-DET can reliably characterize the use of community and healthcare resources and the timing of death relative to the pregnancy and relative to the complication.

Planned place of birth was reliably coded using the M-DET. This is because the narrative contains the families' report of the events as they unfolded. In other studies in which the interviewer asks where the woman delivered, this elicits ‘actual place of birth’ which would typically misclassify data with those who transferred to healthcare facilities for routine care for delivery/abortion, and those who were transported to a health facility for emergency care following complications during a planned home delivery/abortion. Similarly, the direct question “Where was the planned place of birth?” after a maternal death could elicit a form of social desirability bias, since perhaps the family is questioning their decision to have a home birth after an adverse event. By reliably categorizing planned place of birth, home and facility-based births can be separated for analysis and will improve the quality of data for monitoring this indicator.

The identification of the most-responsible-person in labour is meant to differentiate between skilled and unskilled birth attendant – a Millennium Development Goal indicator. The field interviewer needs to be well trained to ascertain professional qualifications of the most-responsible-person. This data extraction tool differentiates and aims to reduce misclassification of the initial most-responsible-person attending the routine delivery/abortion from the most-responsible-person that accepts care of the woman once a complication is identified. Access to the healthcare system via antenatal care, community consultations, transport, and admission and referral to hospital were all reliably extracted using M-DET.

There were areas of data entry errors that would be addressed with a database design versus spread sheet data entry to improve data quality (employing range and consistency checks). Where there was less than perfect agreement for short answer responses (e.g. uptake of antenatal care, delivery by cesarean), data entry error was the most common cause of inter-rater disagreement, as the response was explicit in the short answer of the RHIME and did not require interpretation. Where short answers differed from information provided in the narrative, coders were instructed to code the response in the narrative as the standard - this required interpretation and led to some inter-rater disagreement (e.g. reporting receipt of tetanus toxoid was considered affirmative for receipt of antenatal care, and required some interpretation on the part of the coder). Improved clarification for these short answer interpretation and for data cleaning has been added to the coder's training manual. Our group is in the process of designing an open-access format of the M-DET with the accompanying user's manual. This will be available to interested groups via the Centre for Global Health website (www.cghr.org).

Inter-rater agreement of underlying cause of death was substantial to high for all broad categories of maternal death except for maternal sepsis. Closer analysis of this inter-rater agreement will be presented in an upcoming paper.

When information on maternal deaths is collected as part of a larger verbal autopsy study not specifically designed to study maternal mortality in detail, additional data extraction may be necessary to avoid misclassification of exposures of interest (such as planned place of birth, skill attendance). The M-DET is especially useful to refine the information about events surrounding the relatively rare maternal death (?1% of all deaths in the study) in large scale verbal autopsy studies such as the MDS since it relies mainly on information available in short answers and a written narrative, not requiring the inclusion of additional questions to an existing survey or additional fieldwork training. However it could also be used in conjunction with facility-based maternal death case reviews, facility-based audits or a multiple-source characterization of maternal deaths such as Reproductive Age Mortality Studies [22].

While M-DET is not designed to substitute physician coding of causes of death it could be a useful tool for cause of death quality assurance. Although we recognize that there could be a lack of precision in defining cause of death with verbal autopsies when compared with medical diagnoses, verbal autopsies can provide good estimates of the main causes of pregnancy-related deaths in communities lacking complete vital registration systems and where many women die at home [23]. M-DET obtained moderate to high inter-rater agreement in verifying cause of death (both direct and indirect causes) and a study is underway to measure physician agreement and the quality of the cause of death assignment in comparison with those obtained using the M-DET.
